# Specific genetic markers for detecting subtypes of dengue virus serotype-2 in isolates from the states of Oaxaca and Veracruz, Mexico

**DOI:** 10.1186/1471-2180-8-117

**Published:** 2008-07-15

**Authors:** Catalina E Gardella-Garcia, Gerardo Perez-Ramirez, Joel Navarrete-Espinosa, Alejandro Cisneros, Fabiola Jimenez-Rojas, Luis R Ramírez-Palacios, Rocio Rosado-Leon, Minerva Camacho-Nuez, Maria de L Munoz

**Affiliations:** 1 Department of Genetics and Molecular Biology, Centro de Investigacion y de Estudios Avanzados del Instituto Politecnico Nacional, Av. Instituto Politecnico Nacional 2508, San Pedro Zacatenco, C.P. 07360, Mexico D. F., Mexico; 2 Program of Genomic Sciences, Universidad Autonoma de la Ciudad de Mexico, San Lorenzo #290 Col. Del Valle, Mexico D. F., Mexico; 3 Division de Epidemiologia, Coordinacion de Programas Integrados de Salud, Instituto Mexicano del Seguro Social. Av. Insurgentes Sur 253, 7° Piso, Col. Roma, C.P. 06700 Mexico D. F., Mexico; 4 Escuela de Medicina Veterinaria y Zootecnia, Universidad Autonoma Benito Juarez de Oaxaca, Oaxaca, Mexico; 5 Laboratorio Estatal de Salud Publica de Oaxaca, Servicios de Salud de Oaxaca, Oaxaca, Mexico; 6 Delegacion Veracruz Norte. IMSS, Veracruz, Mexico

## Abstract

**Background:**

Dengue (DEN) is an infectious disease caused by the DEN virus (DENV), which belongs to the *Flavivirus *genus in the family *Flaviviridae*. It has a (+) sense RNA genome and is mainly transmitted to humans by the vector mosquito *Aedes aegypti*. Dengue fever (DF) and dengue hemorrhagic fever (DHF) are caused by one of four closely related virus serotypes (DENV-1, DENV-2, DENV-3 and DENV-4). Epidemiological and evolutionary studies have indicated that host and viral factors are involved in determining disease outcome and have proved the importance of viral genotype in causing severe epidemics. Host immune status and mosquito vectorial capacity are also important influences on the severity of infection. Therefore, an understanding of the relationship between virus variants with altered amino acids and high pathogenicity will provide more information on the molecular epidemiology of DEN. Accordingly, knowledge of the DENV serotypes and genotypes circulating in the latest DEN outbreaks around the world, including Mexico, will contribute to understanding DEN infections.

**Results:**

1. We obtained 88 isolates of DENV, 27 from Oaxaca and 61 from Veracruz. 2. Of these 88 isolates, 16 were serotype 1; 62 serotype 2; 7 serotype 3; and 2 serotype 4. One isolate had 2 serotypes (DENV-2 and -1). 3. Partial nucleotide sequences of the genes encoding C- prM (14 sequences), the NS3 helicase domain (7 sequences), the NS5 S-adenosyl methionine transferase domain (7 sequences) and the RNA-dependent RNA polymerase (RdRp) domain (18 sequences) were obtained. Phylogenetic analysis showed that DENV-2 isolates belonged to the Asian/American genotype. In addition, the Asian/American genotype was divided into two clusters, one containing the isolates from 2001 and the other the isolates from 2005–2006 with high bootstrap support of 94%.

**Conclusion:**

DENV-2 was the predominant serotype in the DF and DHF outbreak from 2005 to 2006 in Oaxaca State as well as in the 2006 outbreak in Veracruz State, with the Asian/American genotype prevalent in both states. Interestingly, DENV-1 and DENV-2 were the only serotypes related to DHF cases. In contrast, DENV-3 and DENV-4 were poorly represented according to epidemiological data reported in Mexico. We found that isoleucine was replaced by valine at residue 106 of protein C in the isolates from these 2005–2006 outbreaks and in those from the 1997, 1998 and 2001 outbreaks in the Caribbean islands. We suggested that this amino acid change may be used as a signature for isolates arising in the Caribbean islands and pertaining to the Asian/American genotype. Other amino acid changes are specific for the Asian/American, Asian and American strains.

## Background

DF and DHF/DSS are mosquito-borne infectious diseases transmitted by the vector *Ae. Aegypti *and have become a major global health concern. This mosquito vector is endemic in several regions of Mexico, especially the South Pacific Coast [1-3]. DF and DHF/DSS occur in tropical and sub-tropical regions around the world, predominantly in urban and suburban areas. The illness is endemic in more than 100 cities in Africa, America, the Mediterranean region and Southeast Asia [4]. Fifty million people are infected every year; approximately 500,000 of them are hospitalized and between 5% and 15% die [5]. This viral illness is characterized by a sudden onset of symptoms including high fever, severe headaches, extreme myalgias and arthralgias, retro-orbital pain, anorexia, gastrointestinal disturbances, exanthema, nausea and vomiting; a rash may appear three or four days after the fever begins [6,7].

The infection caused by any of the four serotypes of DENV confers specific permanent immunity to the virus but does not protect against the other serotypes [8]; however, short-term cross-immunity against the other three serotypes has been reported [9]. People living in an endemic area can be infected with any of the four serotypes of DENV and can even be simultaneously infected by two serotypes [10]. The relevance to pathology of the serotype and genotype of the infecting strain has been described several times [11-13].

The increase in the number of DHF and DSS cases worldwide is not fully understood. It was first explained by mutations making DENV more virulent, but this hypothesis has not been supported. Then, the "secondary infection" or "immune enhancement" hypothesis was proposed to explain the increased virulence of DENV in patients with secondary infections. This hypothesis is still favoured [14]. However, observations in Southeast Asia, some Pacific islands and the Americas are not consistent with it, so it has been modified several times. Advances in molecular biology have led to the recognition that some viral strains are more virulent than others [11-13]. The introduction of DHF into Cuba in 1981 was linked to the arrival of a Southeast Asian strain of DENV-2 on the island [15]. Subsequently, an extended outbreak of DHF/DSS occurred in Venezuela and Mexico, and this was linked to the introduction of the same Southeast Asian strain of DENV-2 [16,17]. Furthermore, a major epidemic of DF due to DENV-2 with the American genotype was reported in Peru in 1995, about 5 years after an epidemic of DENV-1 [18]. The American DENV-2 genotype strains may have lacked the properties necessary to cause severe disease. The American genotype has been associated with mild disease (DF), while the Southeast Asian genotype coincided with the appearance of DHF on the American continent [11]. On this basis, viral virulence and immune responses have been considered two major factors in the pathogenesis of DHF.

Early phylogenetic studies organized DENV sequences into clusters on trees and classified them into a series of genotypes or subtypes [19-22]. Within DENV-2, six genotypes have been proposed, five of which occur in humans [22,23]. Although the Asian/American genotype originated in Southeast Asia and spread to the Americas in the early 1980s [22], the strains phylogenetically classified as Asian I and Asian II are currently restricted to Southeast Asia [24]; the American genotype is mostly found in the Americas and its frequency is now decreasing; and the Cosmopolitan genotype is widely distributed across the tropical and subtropical world [24,25]. DENV-1 consists of five subtypes (I-V) [22]. The DENV-3 and -4 viruses are currently classified into four and two genotypes, respectively [19,20].

As mentioned above, DENV genotypes differ in virulence, including their human pathogenicities and epidemic potential. Therefore, phylogenetic studies were conducted to determine the epidemic potential of DENV isolates from Veracruz and Oaxaca. These studies were carried out using partial sequences of the genes encoding C-prM, NS5 and NS3. C and prM were chosen because they represent a region that is commonly analyzed in studies of the molecular epidemiology of DEN [17,26]. NS5 and NS3 are important because they may be useful for determining the genotype and may help us to establish additional specific genetic markers. Also, NS5 has more nucleotide substitutions [27] and five of the eight amino acid differences between the American and Asian genotypes are located in the N terminus of this protein [12]. The RdRp of NS5 is crucial for viral RNA replication. NS5 also contains an MTase domain that is essential for the interaction with RdRp required for virus replication and/or infectivity [28]. In addition, *NS5 *has been used thoroughly in studies of flavivirus phylogenies [29,30].

The interdomain (linker) region 169–179 of NS3 and the two loops that encircle the entrance of the ATP binding pocket [31] were also studied because of the role of this protein in virus replication. This region has also been implicated in the intrinsic flexibility that couples movements between the NS3 protease and helicase domains [32].

In summary, phylogenetic and genetic analyses of Oaxaca and Veracuz virus isolates reveal the putative geographic origins of the viruses and potential molecular determinants of virulence.

## Results

### Virus isolation and serotyping

**Figure 1:**
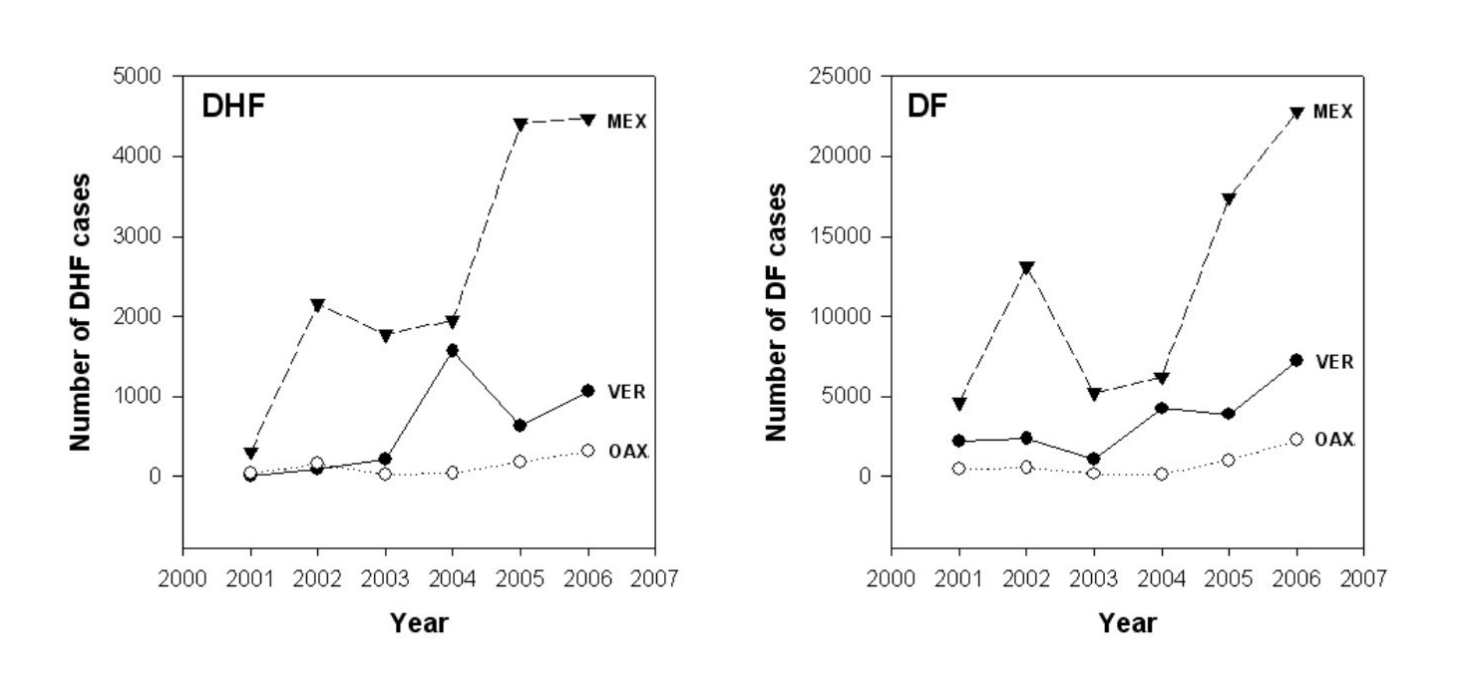
**Epidemiology of DEN in Mexico (2000–2006)**. The numbers of DF and DHF cases were obtained from CENAVECE [34]. The total numbers of DF and DHF cases in Oaxaca and Veracruz states are compared with the numbers in Mexico as a whole.

The Mac-Elisa test has been implemented by the Ministry of Health in Mexico for diagnosing DENV infections [33]. Patients whose clinical reports show symptoms of DHF are exclusively evaluated using an IgG-ELISA kit. Reported cases are under-notified and the circulating serotype is not widely known. Analysis of the circulating serotype is important since it has been observed that DEN cases in Mexico are increasing annually (Figure 1). This Figure also shows that the percentage of DHF cases has increased in comparison to DF cases [34].

**Figure 2:**
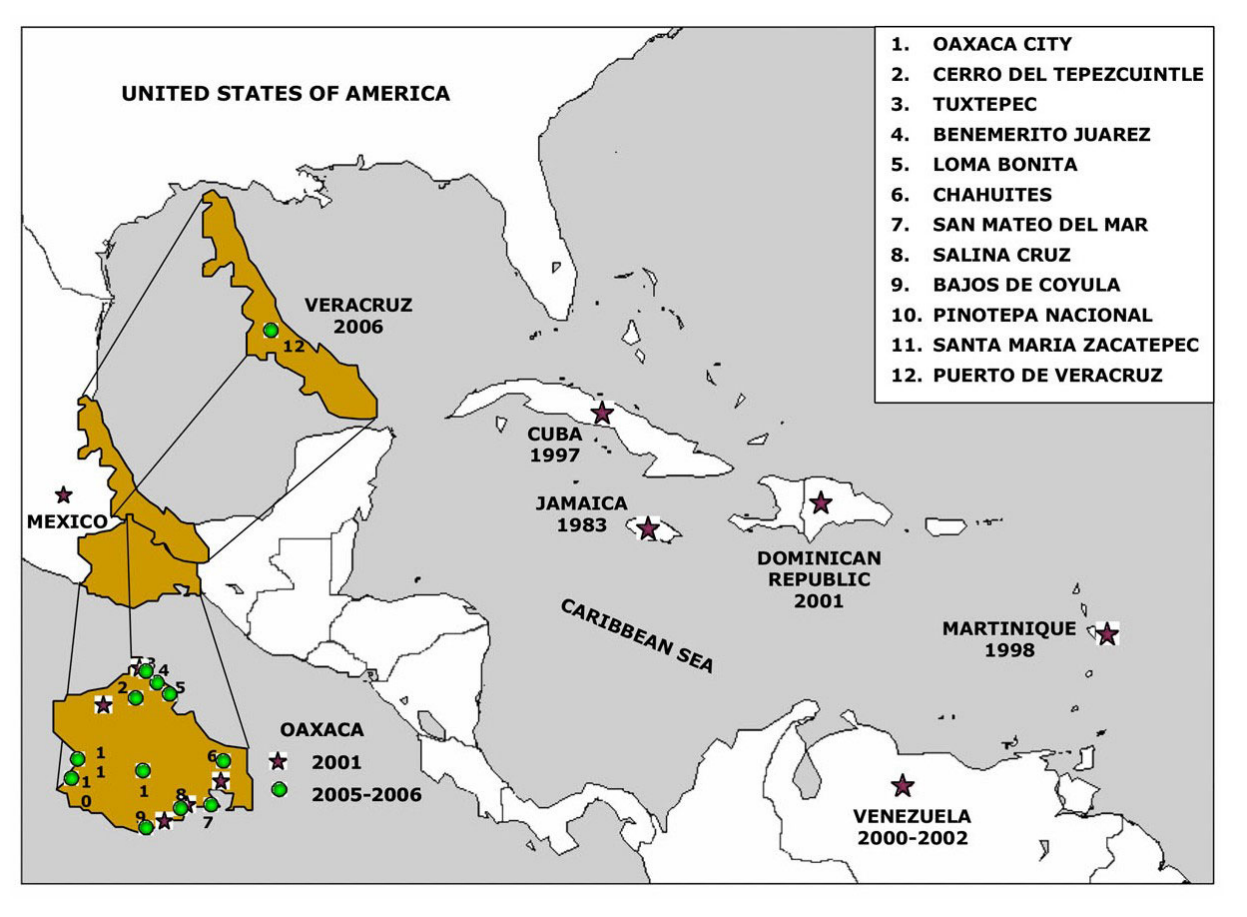
**Mexican and Caribbean region map**. The virus sample collection sites for the 13 isolates that were sequenced from Oaxaca and Veracruz States are shown in addition to the sites of origin of the GenBank sequences from the strains used in this phylogenetic study. These strains are from Caribbean countries and are grouped in the same branch as the sequences of our isolates corresponding to the Asian/American genotype.

**Figure 3:**
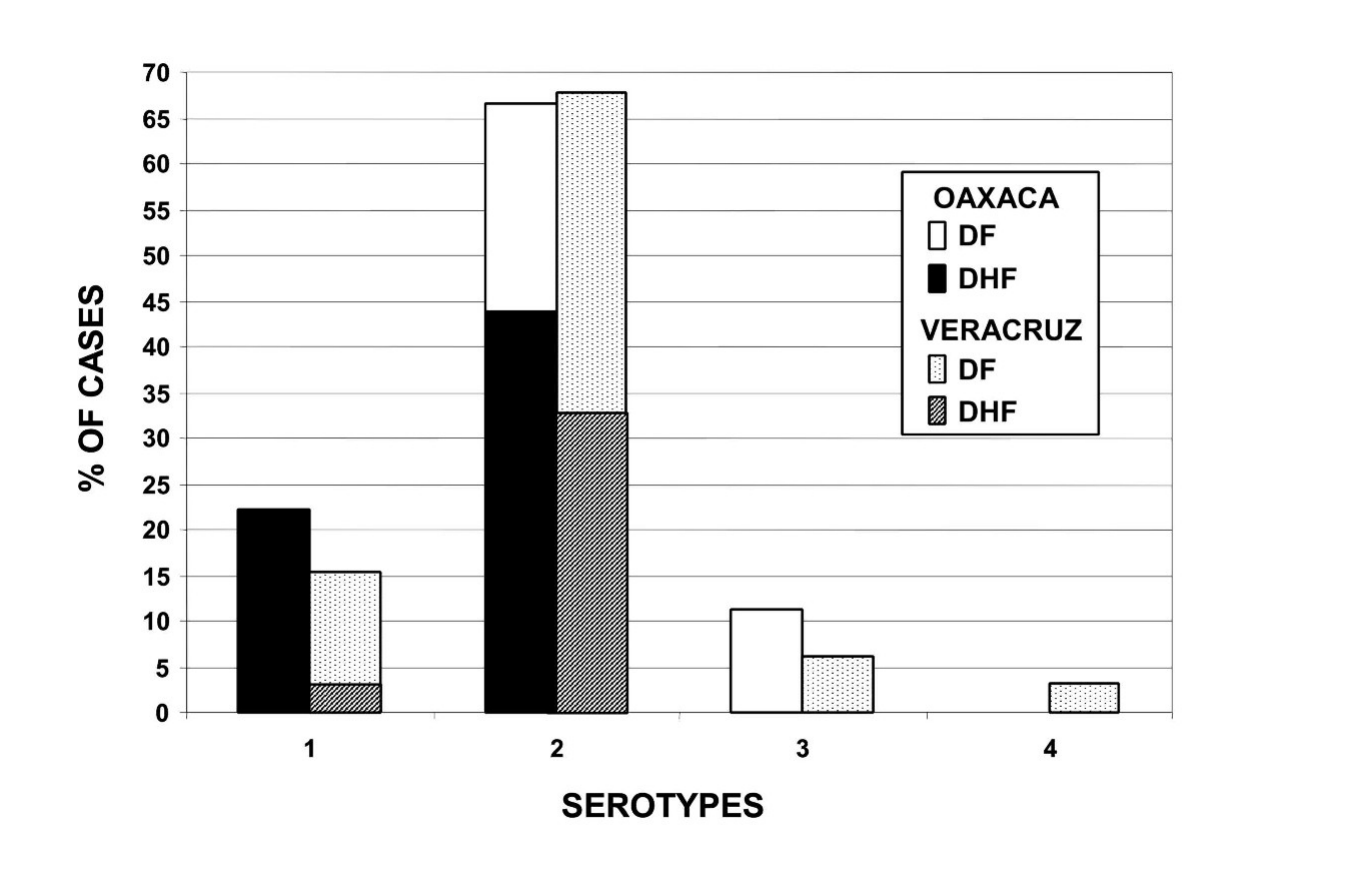
**Summary of all DENV analyzed in this study**. The percentages of DENV-1, 2, 3 and 4 detected in Oaxaca and Veracruz States during outbreaks in 2005–06. The graph shows that the relative percentages of DENV-1, 2, 3 and 4 do not differ between Oaxaca and Veracruz, although the numbers of samples analyzed were different.

The serotypes of 88 DENV samples from different sites in Oaxaca and Veracruz States, Mexico (Figure 2) were evaluated and the results are shown in Figure 3. Serotyping was performed by RT-PCR using the RNA obtained from isolates in C6/36 cells; each test to determine serotype was repeated at least twice. C6/36 cells were chosen because the nucleotide sequence of the virus genome is conserved [35,36].

Out of 27 isolates from Oaxaca, 18 were diagnosed with DHF; 12 were serotype 2 and six were serotype 1 (Table 1). Among the 61 isolates obtained from patient sera in Veracruz State, fifteen with DHF were serotype 2, two were serotype 1 and the isolate VER/MEX013A/06 showed both serotypes 1 and 2.

**Table 1:** Serotype determination of isolates from Oaxaca (2005–2006) and Veracruz (2006).

**SEROTYPE**	**DIAGNOSIS**	**OAXACA**	**VERACRUZ**
**1**	DHF	**6**	**2**
	DF	**0**	**8**
**2**	DHF	**12**	**15**
	DF	**6**	**29**
**3**	DF	**3**	**4**
**4**	DF	**0**	**2**
**1,2**	DHF	**0**	**1**

Out of a total of 52 isolates with DF, 35 were serotype 2, seven serotype 3, eight serotype 1 and two serotype 4 (Table 1). Known strains of DENV-1, -2, -3 and -4 were used as positive controls for the RT-PCR assays.

Figure 3 shows the percentages of cases of DENV-1 (22%, 16%), DENV-2 (67%, 72%) and DENV-3 (11%, 6%) in Oaxaca and Veracruz respectively; DENV-4 cases had the lowest percentages (0%, 3%). These values were consistent in both States.

### Phylogenetic analysis of Oaxaca isolates

Portions of the C_91_-prM_1_, NS5_73_, NS5_2206 _and NS3_484 _genes were amplified and sequenced as described in the Methods section. Fourteen sequences were obtained from 8 isolates from Oaxaca and 6 from Veracruz for the C-prM fragment (11 with DF and 3 with DHF); 7 sequences were obtained from 5 Oaxaca isolates and 2 from Veracruz isolates for the MTase domain (NS5_73_) fragment (4 with DF and 3 with DHF); 18 sequences were obtained from 7 Oaxaca isolates and 11 from Veracruz isolates for the RdRp domain (NS5_73_) fragment (12 with DF and 6 with DHF); 7 sequences were obtained from 7 Oaxaca isolates for the helicase domain fragment (6 with DF and 1 with DHF).

Phylogenetic analysis was then performed on these sequences, including other prototype sequences of characterized isolates for DENV-2 (Table 2, 3).

**Table 2:** Summary of consistent amino acid change among the five DENV-2 genotypes viruses.

PROTEIN				AMINO ACID POSITIONS^a^										
C and PrM				**101**	**104**	**106**	**108**	**112**	**129**	**130**	**142**	**143**	**145**	**153**

PrM									**14**	**15**	**28**	**29**	**31**	**39**

**STRAIN/YEAR**	**ACCESSION GENBANK No**.	**DIAGNOSIS**	**GENOTYPE**											

Tonga/74	AY744147	DF	AMERICAN	T	M	I	L	V	S	R	**K**	D	**T**	M
S1vaccine/USA/93	M19197	DF	AMERICAN	T	M	I	L	V	S	R	**K**	D	**T**	M
131/MEX/92	AF100469	DF	AMERICAN	T	M	I	L	V	S	R	**K**	D	**T**	M
I348600/COL/86	AY702040	DF	AMERICAN	T	M	I	L	V	S	R	**K**	D	**T**	M
P23085 INDI-60/01	DQ448238	DF	AMERICAN	T	M	I	L	V	S	R	**K**	D	**T**	M
Ven2/87	AF100465	DF	AMERICAN	T	M	I	L	V	S	R	**K**	D	**T**	**L**
HUAT2/MEX/01	AY692469	DF	ASIAN/AMERICAN	T	**V**	I	L	**A**	**G**	R	**K**	V	V	**L**
SALC9/MEX/01	AY692465	DF	ASIAN/AMERICAN	T	**V**	I	L	**A**	**G**	R	**K**	D	V	**I**
HUAT11/MEX/01	AY692470	DHF	ASIAN/AMERICAN	T	**V**	I	L	**A**	**G**	R	**K**	D	V	**I**
HUAT12/MEX/01	AY692471	DF	ASIAN/AMERICAN	T	**V**	I	L	**A**	**G**	R	E	D	V	**I**
TUX19/MEX/01	AY692467	DHF	ASIAN/AMERICAN	T	**V**	I	L	**A**	**G**	R	E	D	V	**I**
TON4/MEX/01	AY692468	DHF	ASIAN/AMERICAN	T	**V**	I	L	**A**	**G**	R	E	D	V	**I**
JUCH5/MEX/01	AY692466	DHF	ASIAN/AMERICAN	T	**V**	I	L	**A**	**G**	R	E	D	V	**I**
OAX/MEXS707/05*	EF595822	DF	ASIAN/AMERICAN	T	**V**	**V**	L	**A**	**G**	R	E	D	V	M
OAX/MEXS1038/05*	EF595828	DF	ASIAN/AMERICAN	T	**V**	**V**	L	**A**	**G**	R	E	D	V	**I**
OAXS1656/MEX/05*	EF595834	DF	ASIAN/AMERICAN	T	**V**	**V**	L	**A**	**G**	R	E	D	V	**I**
OAX/MEXS739/05*	EF595829	DF	ASIAN/AMERICAN	T	**V**	**V**	L	**A**	**G**	R	E	D	V	**I**
OAX/MEXS1733/06*	EF595821	DF	ASIAN/AMERICAN	T	**V**	**V**	L	**A**	**G**	R	E	D	V	**I**
OAX/MEXS14946/06*	EF595800	DHF	ASIAN/AMERICAN	T	**V**	**V**	L	**A**	**G**	R	E	D	V	**I**
OAX/MEXS14760/06*	EF595824	DHF	ASIAN/AMERICAN	T	**V**	**V**	L	**A**	**G**	R	E	D	V	**I**
OAX/MEXS1020/06*	EF595823	DHF	ASIAN/AMERICAN	T	**V**	**V**	L	**A**	**G**	R	E	D	V	**I**
VER/MEXS013A/06*	EU552535	DHF	ASIAN/AMERICAN	T	**V**	**V**	L	**A**	**G**	R	E	D	V	**I**
VER/MEXS011A/06*	EU552536	DHF	ASIAN/AMERICAN	T	**V**	**V**	L	**A**	**G**	R	E	D	V	**I**
VER/MEXS008A/06*	EU552538	DHF	ASIAN/AMERICAN	T	**V**	**V**	L	**A**	**G**	R	E	D	V	**I**
VER/MEXS015A/06*	EU552534	DF	ASIAN/AMERICAN	T	**V**	**V**	L	**A**	**G**	R	E	D	V	**I**
VER/MEXS033A/06*	EU552537	DF	ASIAN/AMERICAN	T	**V**	**V**	L	**A**	**G**	R	E	D	V	**I**
VER/MEXS020A/O6*	EU552539	DF	ASIAN/AMERICAN	T	**V**	**V**	L	**A**	**G**	R	E	D	V	**I**
Cuba13/97	AY702034	DF	ASIAN/AMERICAN	T	**V**	**V**	L	**A**	**G**	R	E	D	V	**I**
Cuba115/97	AY702036	DF	ASIAN/AMERICAN	T	**V**	**V**	L	**A**	**G**	R	E	D	V	**I**
Cuba205/97	AY702039	DSS	ASIAN/AMERICAN	T	**V**	**V**	L	**A**	**G**	R	E	D	V	**I**
Cuba165/97	AY702038	DHF	ASIAN/AMERICAN	T	**V**	**V**	L	**A**	**G**	R	E	D	V	**I**
Cuba58/97	AY702035	DF	ASIAN/AMERICAN	T	**V**	**V**	L	**A**	**G**	R	E	D	V	**I**
Cuba89/97	AY702037	DHF/DSS	ASIAN/AMERICAN	T	**V**	**V**	L	**A**	**G**	R	E	D	V	**I**
MART/98-703/98	AF208496	DF	ASIAN/AMERICAN	T	**V**	**V**	L	**A**	**G**	R	E	D	V	**I**
DR23/01	AB122020	DF	ASIAN/AMERICAN	T	**V**	**V**	L	**A**	**G**	R	E	D	V	**I**
DR31/01	AB122021	DF	ASIAN/AMERICAN	T	**V**	**V**	L	**A**	**G**	R	E	D	V	**I**
DR59/01	AB122022	DHF	ASIAN/AMERICAN	T	**V**	**V**	L	**A**	**G**	R	E	D	V	**I**
LARD1701/Ven/01	AF360861	DF	ASIAN/AMERICAN	T	**V**	**V**	L	**A**	**G**	R	E	D	V	**I**
LARD1432/Ven/01	AF360860	DF	ASIAN/AMERICAN	T	**V**	**V**	L	**A**	**G**	R	E	D	V	**I**
LARD1910/Ven/01	AF360862	DF	ASIAN/AMERICAN	T	**V**	I	L	**A**	**G**	R	E	D	V	**I**
LARD1996/Ven/01	AF360863	DF	ASIAN/AMERICAN	T	**V**	I	L	**A**	**G**	R	E	D	V	**I**
Mara3/Ven/02	AY0444421	DHF	ASIAN/AMERICAN	T	**V**	I	L	**A**	**G**	R	E	D	V	**I**
BR64022/02	AF489932	NA	ASIAN/AMERICAN	T	**V**	I	L	**A**	**G**	R	E	D	V	**I**
Jamaica/1409/83	M20558	DHF	ASIAN/AMERICAN	T	**V**	I	L	**A**	**G**	R	E	D	V	**I**
China 04	U87411	DHF	ASIAN/AMERICAN	T	**V**	I	L	**A**	**G**	R	E	D	V	**I**
CO390/Th/99	AF100462	DHF	ASIAN 1	**S**	**V**	I	L	V	S	R	E	D	V	M
Bangkok 1974	AJ487271	NA	ASIAN 1	**S**	M	I	L	V	S	R	E	D	V	M
16681/USA/97	U87411	NA	ASIAN 1	**S**	M	I	L	V	S	R	E	V	V	M
PDK53/USA/97	U87412	NA	ASIAN 1	**S**	M	I	L	V	S	R	E	V	V	M
16681-PDK53/97	U87411	DHF	ASIAN 1	**S**	M	I	L	V	S	R	E	D	V	M
ThNH-p11/93	AF022437	DF	ASIAN 1	**S**	M	I	L	V	S	R	E	D	V	M
ThNH-p12/93	AF022438	DF	ASIAN 1	**S**	M	I	L	V	S	R	E	D	V	M
ThNH-p14/93	AF022439	DHF	ASIAN 1	**S**	M	I	L	V	S	R	E	D	V	M
ThNH-7/93	AF022434	DSS	ASIAN 1	**S**	M	I	L	V	S	**I**	E	D	V	M
ThNH-28/93	AF022435	DHF	ASIAN 1	**S**	M	I	L	V	S	**I**	E	D	V	M
ThNH-52/93	AF022436	DHF	ASIAN 1	**S**	M	I	L	V	S	**I**	E	D	V	M
ThNH-p16/93	AF022440	DF	ASIAN 1	**S**	M	I	L	V	S	**I**	E	D	V	M
ThNH-p36/93	AF022441	DHF	ASIAN 1	**S**	M	I	L	V	S	**I**	E	D	V	M
ThNH29/93	AF169678	DHF	ASIAN 1	**S**	M	I	L	V	S	**I**	E	D	V	M
ThNH36/93	AF169679	DF	ASIAN 1	**S**	M	I	L	V	S	**I**	E	D	V	M
ThNH45/93	AF169680	DHF	ASIAN 1	**S**	M	I	L	V	S	**I**	E	D	V	M
ThNH55/93	AF169681	DHF	ASIAN 1	**S**	M	I	L	V	S	**I**	E	D	V	M
ThNH54/93	AF169682	DHF	ASIAN 1	**S**	M	I	L	V	S	**I**	E	D	V	M
ThNH62/93	AF169683	DHF	ASIAN 1	**S**	M	I	L	V	S	**I**	E	D	V	M
ThNH63/93	AF169684	DHF	ASIAN 1	**S**	M	I	L	V	S	**I**	E	D	V	M
ThNH69/93	AF169685	DHF	ASIAN 1	**S**	M	I	L	V	S	**I**	E	D	V	M
ThNH73/93	AF169686	DHF	ASIAN 1	**S**	M	I	L	V	S	**I**	E	D	V	M
ThNH76/93	AF169687	DHF	ASIAN 1	**S**	M	I	L	V	S	**I**	E	D	V	M
ThNH81/93	AF169688	DHF	ASIAN 1	**S**	M	I	L	V	S	**I**	E	D	V	M
Mara 4/Ven/90^&^	AF100466	DHF	ASIAN 1	**S**	M	I	L	V	S	**I**	E	D	V	M
K0008/Th/99	AF100459	DHF	ASIAN 1	**S**	M	I	L	V	S	**I**	E	D	V	M
K0010/Th/99	AF100460	DF	ASIAN 1	**S**	M	I	L	V	S	**I**	E	D	V	M
C0371/Th/99	AF100461	DF	ASIAN 1	**S**	M	I	L	V	S	**I**	E	D	V	M
C0166/Th/99	AF100463	DF	ASIAN 1	**S**	M	I	L	V	S	**I**	E	D	V	M
C0167/Th/99	AF100464	DHF	ASIAN 1	**S**	M	I	L	V	S	**I**	E	D	V	M
New Guinea C/44	D00346	DHF	ASIAN 2	T	M	I	L	V	S	R	E	D	V	M
43/China/89	AF204178	DF	ASIAN 2	T	M	I	L	V	S	R	G	D	V	M
44/China/87	AF204177	DF	ASIAN 2	T	M	I	L	V	S	R	G	D	V	M
BA05i/Indon/05	AY858035	DF	COSMOPOLITAN	T	I	I	**M**	V	S	R	E	**N**	V	M
TSV01/Austra/93	AY037116	NA	COSMOPOLITAN	T	I	I	**M**	V	S	R	E	**N**	V	M
98900666/Indo/04	AB189124	DSS	COSMOPOLITAN	T	**V**	I	**M**	V	S	R	E	**N**	V	M
ZS01/China/01	EF051521	NA	COSMOPOLITAN	T	**V**	I	**M**	V	S	R	E	**N**	V	M
GWL18/INDI/01	DQ448231	NA	COSMOPOLITAN	T	**V**	I	L	**A**	S	R	E	D	V	M
GWL39/INDI/01	DQ448232	NA	COSMOPOLITAN	T	**V**	I	L	**A**	S	R	E	D	V	M

^a^Amino acid sequence was numbered starting from protein C or prM to the end of the last codon sequenced. *Sequences of this study.

^&^Recombinant strain [16].

**Table 3:** Summary of consistent amino acid change among the five DENV-2 genotype viruses.

**PROTEIN**				**AMINO ACID POSITIONS^A^**															
	
			**DOMAIN**	**MTase**					**RdRp**								**Helicase †**		
**NS5**				**026**	**155**	**179**	**192**		**748**	**762**	**784**	**799**	**810**	**818**	**835**	**864**			

**NS3**																	**187**	**249**	**250**

**STRAIN/YEAR**	**ACCESSION GENBANK**	**DIAGNOSIS**	**GENOTYPE**																

Tonga/74	AY744147	DF	AMERICAN	I	V	**V**	**R**		R	S	S	**S**	T	**L**	I	T	R	R	A
S1 vaccine/USA/93	M19197	NA	AMERICAN	I	V	**V**	**R**		R	S	S	**S**	T	**L**	I	T	R	R	A
131/MEX/92	AF100469	DF	AMERICAN	I	V	**V**	**R**		R	S	S	**S**	T	**L**	I	T	R	R	A
Ven2/87	AF100465	DF	AMERICAN	I	V	**V**	**R**		R	S	S	**S**	T	**L**	I	T	R	R	A
I348600/COL/86	AY702040	DF	AMERICAN	I	V	**V**	**R**		R	S	S	**S**	T	**L**	I	T	R	R	A
OAX/MEX/S707/05^§^	EF595821	DF	ASIAN/AMERICAN	**-**	**-**	-	-		**K**	**T**	**P**	K	**A**	Q	**V**	T	**K**	**K**	**T**
OAX/MEXS1038/05^§^	EF595817/EF595825	DF	ASIAN/AMERICAN	**V**	**I**	I	K		**K**	**T**	**P**	K	**A**	Q	**V**	T	**K**	**K**	**T**
OAX/MEXS1656/05^§^	EF595815/EF595820	DF	ASIAN/AMERICAN	**V**	**I**	I	K		**K**	**T**	**P**	K	**A**	Q	**V**	T	**K**	**K**	**T**
OAX/MEXS739/05^§^	EF595818/EF595822	DF	ASIAN/AMERICAN	**V**	**I**	I	K		**K**	**T**	**P**	K	**A**	Q	**V**	T	**K**	**K**	**T**
OAX/MEXS1020/06^§^	EF595816/EF595824	DHF	ASIAN/AMERICAN	**V**	**I**	I	K		**K**	**T**	**P**	K	**A**	Q	**V**	T	**K**	**K**	**T**
OAX/MEXS1733/06^§^	EF595819/EF595823	DF	ASIAN/AMERICAN	**V**	**I**	I	K		**K**	**T**	**P**	K	**A**	Q	**V**	T	**K**	**K**	**T**
VER/MEX024A/06^§^	EU570975/EU570983	DHF	ASIAN/AMERICAN	**V**	**I**	I	K		**K**	**T**	**P**	K	**A**	Q	**V**	T	-	-	-
VER/MEX013A/06^§^	EU570980/EU570982	DHF	ASIAN/AMERICAN	**V**	**I**	I	K		**K**	**T**	**P**	K	**A**	Q	**V**	T	-	-	-
Cuba13/97	AY702034	DF	ASIAN/AMERICAN	I	**I**	I	K		**K**	**T**	S	K	**A**	Q	**V**	T	**K**	R	A
Cuba115/97	AY702036	DF	ASIAN/AMERICAN	I	**I**	I	K		**K**	**T**	S	K	**A**	Q	**V**	T	**K**	R	A
Cuba205/97	AY702039	DSS	ASIAN/AMERICAN	I	**I**	I	K		**K**	**T**	S	K	**A**	Q	**V**	T	**K**	R	A
Cuba165/97	AY702038	DHF	ASIAN/AMERICAN	I	**I**	I	K		**K**	**T**	S	K	**A**	Q	**V**	T	**K**	R	A
Cuba58/97	AY702035	DF	ASIAN/AMERICAN	I	**I**	I	K		**K**	**T**	S	K	**A**	Q	**V**	T	**K**	R	A
Cuba89/97	AY702037	DHF/DSS	ASIAN/AMERICAN	I	**I**	I	K		**K**	**T**	S	K	**A**	Q	**V**	T	**K**	R	A
MART/98-703/98	AF208496	DF	ASIAN/AMERICAN	I	**I**	I	K		**K**	**T**	S	K	**A**	Q	**V**	T	**K**	R	A
DR23/01	AB122020	DF	ASIAN/AMERICAN	I	**I**	I	K		**K**	**T**	S	K	**A**	Q	**V**	T	**K**	R	A
DR31/01	AB122021	DF	ASIAN/AMERICAN	I	**I**	I	K		**K**	**T**	S	K	**A**	Q	**V**	T	**K**	R	A
DR59/01	AB122022	DHF	ASIAN/AMERICAN	I	**I**	I	K		**K**	**T**	S	K	**A**	Q	**V**	T	**K**	R	A
BR64022/02	AF489932	NA	ASIAN/AMERICAN	I	**I**	I	K		**K**	**T**	S	K	**A**	Q	**V**	T	**K**	R	A
Mara 4^&^/Ven/90	AF100466	DHF	ASIAN/AMERICAN	I	**I**	I	K		**K**	**T**	S	K	**A**	Q	I	T	**K**	R	A
Jamaica/N.1409/83	M20558	NA	ASIAN/AMERICAN	I	**I**	I	K		**K**	**T**	S	K	**A**	Q	**V**	T	**K**	R	A
China 04/85	AF119661	DHF	ASIAN/AMERICAN	I	**I**	I	K		**K**	**S**	S	K	T	Q	**V**	T	**K**	R	**T**
Bangkok 1974	AJ487271	NA	ASIAN 1	I	V	I	K		R	**T**	S	K	T	Q	I	**A**	R	R	A
CO390/Th/99	AF100462	DHF	ASIAN 1	I	V	I	K		R	S	S	K	T	Q	I	**A**	R	R	A
16681/USA/97	U87411	NA	ASIAN 1	I	V	I	K		R	S	S	K	T	Q	I	**A**	R	R	A
PDK53/USA/97	U87412	NA	ASIAN 1	I	V	I	K		R	S	S	K	T	Q	I	**A**	R	R	A
16681-PDK53/97	U87411	DHF	ASIAN 1	I	V	I	K		R	S	S	K	T	Q	I	**A**	R	R	A
ThNH-p11/93	AF022437	DF	ASIAN 1	I	V	I	K		R	S	S	K	T	Q	I	**A**	R	R	A
ThNH-p12/93	AF022438	DF	ASIAN 1	I	V	I	K		R	S	S	K	T	Q	I	**A**	R	R	A
ThNH-p14/93	AF022439	DHF	ASIAN 1	I	V	I	K		R	S	S	K	T	Q	I	**A**	R	R	A
ThNH-7/93	AF022434	DSS	ASIAN 1	I	V	I	K		R	S	S	K	T	Q	I	**A**	R	R	A
ThNH-28/93	AF022435	DHF	ASIAN 1	I	V	I	K		R	S	S	K	T	Q	I	**A**	R	R	A
ThNH-52/93	AF022436	DHF	ASIAN 1	I	V	I	K		R	S	S	K	T	Q	I	**A**	R	R	A
ThNH-p16/93	AF022440	DF	ASIAN 1	I	V	I	K		R	S	S	K	T	Q	I	**A**	R	R	A
ThNH-p36/93	AF022441	DHF	ASIAN 1	I	V	I	K		R	S	S	K	T	Q	I	**A**	R	R	A
ThNH29/93	AF169678	DHF	ASIAN 1	I	V	I	K		R	S	S	K	T	Q	I	**A**	R	R	A
ThNH36/93	AF169679	DF	ASIAN 1	I	V	I	K		R	S	S	K	T	Q	I	**A**	R	R	A
ThNH45/93	AF169680	DHF	ASIAN 1	I	V	I	K		R	S	S	K	T	Q	I	**A**	R	R	A
ThNH55/93	AF169681	DHF	ASIAN 1	I	V	I	K		R	S	S	K	T	Q	I	**A**	R	R	A
ThNH54/93	AF169682	DHF	ASIAN 1	I	V	I	K		R	S	S	K	T	Q	I	**A**	R	R	A
ThNH62/93	AF169683	DHF	ASIAN 1	I	V	I	K		R	S	S	K	T	Q	I	**A**	R	R	A
ThNH63/93	AF169684	DHF	ASIAN 1	I	V	I	K		R	S	S	K	T	Q	I	**A**	R	R	A
ThNH69/93	AF169685	DHF	ASIAN 1	I	V	I	K		R	S	S	K	T	Q	I	**A**	R	R	A
ThNH73/93	AF169686	DHF	ASIAN 1	I	V	I	K		R	S	S	K	T	Q	I	**A**	R	R	A
ThNH76/93	AF169687	DHF	ASIAN 1	I	V	I	K		R	S	S	K	T	Q	I	**A**	R	R	A
ThNH81/93	AF169688	DHF	ASIAN 1	I	V	I	K		R	S	S	K	T	Q	I	**A**	R	R	A
K0008/Th/99	AF100459	DHF	ASIAN 1	I	V	I	K		R	S	S	K	T	Q	I	**A**	R	R	A
K0010/Th/99	AF100460	DF	ASIAN 1	I	V	I	K		R	S	S	K	T	Q	I	**A**	R	R	A
C0371/Th/99	AF100461	DF	ASIAN 1	I	V	I	K		R	S	S	K	T	Q	I	**A**	R	R	A
C0166/Th/99	AF100463	DF	ASIAN 1	I	V	I	K		R	S	S	K	T	Q	I	**A**	R	R	A
New Guinea C/44	D00346	DHF	ASIAN 2	I	V	I	K		R	S	S	K	T	Q	I	T	R	R	A
43/China/89	AF204178	DF	ASIAN 2	I	V	I	K		R	S	S	K	T	Q	I	T	R	R	A
44/China/87	AF204177	DF	ASIAN 2	I	V	I	K		R	S	S	K	T	Q	I	T	R	R	A
BA05i/Indon/05	AY858035	DF	COSMOPOLITAN	I	V	I	K		R	S	S	T	T	Q	I	T	**K**	R	A
TSV01/Austra/93	AY037116	NA	COSMOPOLITAN	I	V	I	K		R	S	S	T	T	Q	I	T	**K**	R	A
98900666/Indo/04	AB189124	DSS	COSMOPOLITAN	I	V	I	K		R	S	S	T	T	Q	I	T	**K**	R	A
FJ-10/China/00	AF276619	NA	COSMOPOLITAN	I	V	I	K		R	S	S	R	T	Q	I	T	**K**	R	A

^&^Amino acid sequence was numbered starting from protein NS5 or NS3 to the end of the last codon sequenced.

*Recombinant strain [16].

^†^NS3 Accession number for Oaxaca isolates: EF591287–EF591293.

^§^Accession numbers for SAM/RdRp Domains

**Figure 4:**
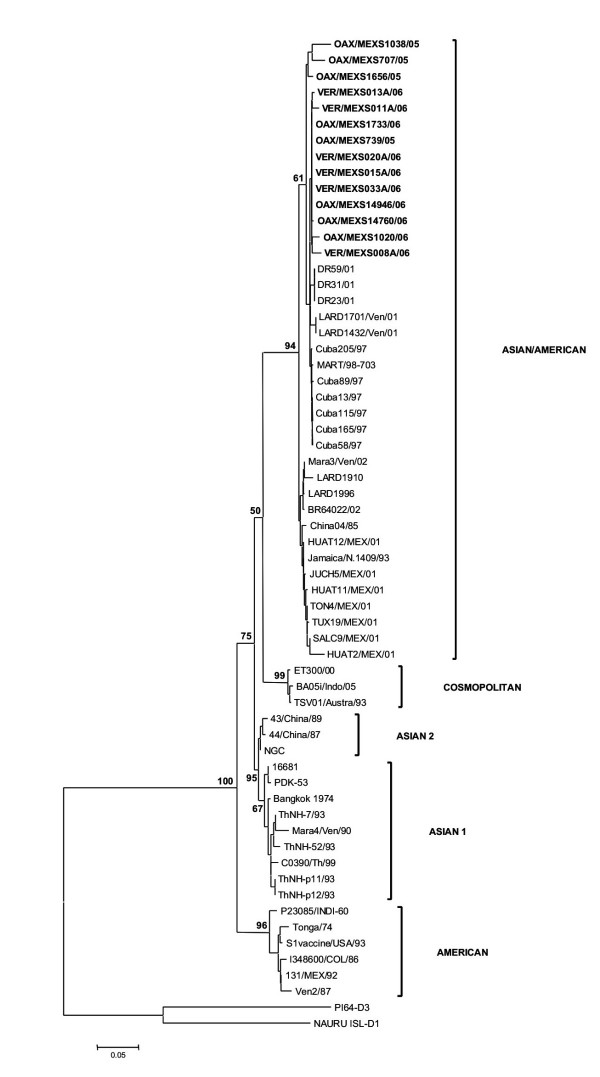
**Minimal evolutionary tree of DENV-2 derived from the C_91_-prM_1 _gene**. Branch lengths are proportional to percentage divergence. For distance/neighbor joining, maximum likelihood and Tamura Nei analyses, 1000 bootstrap replications were performed with MEGA 4.0 [55]. The percentage of replications supporting each branch, by parsimony analysis with gaps included, appears below the relevant branch. Oaxaca isolates appear in bold in the Asian/American genotype cluster. DENV-1, 3 and 4 were used as outgroups.

This analysis showed that C_91_-prM_1_, NS5_75 _and NS3_484 _in the DENV-2 isolates from Oaxaca and Veracruz were most closely related to strains from the Caribbean islands Cuba, Dominican Republic and Martinique (Figure 4). The phylogenetic tree for NS5_75 _and NS3_484 _is not shown. The strains from Brazil, Venezuela and Jamaica formed an independent clade. Interestingly, analysis of *C*_91_*-prM*_1 _showed that the isolates obtained in 2001 belonged to a different clade from those obtained in 2005–2006, with a high bootstrap support of 94%.

**Figure 5:**
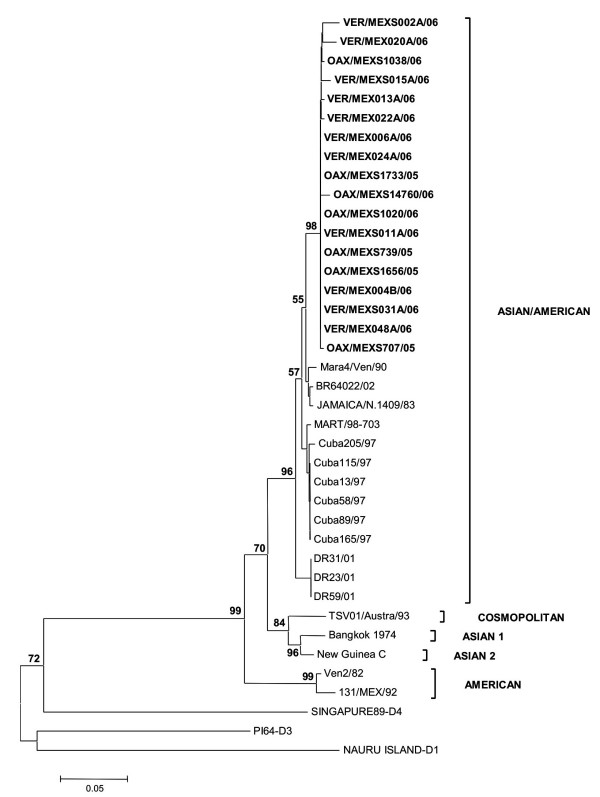
**Minimal evolutional tree of DENV-2 derived from NS5_2206 _gene**. The tree was constructed as described in Figure 4. The isolates from Veracruz and Oaxaca clustered in the Asian/American genotype. DENV-1, 3 and 4 were used as outgroups.

Phylogenetic analysis of *NS5*_2206 _gave a somewhat different pattern: essentially, the isolates belonged to the same clade as those from Venezuela, Brazil and Jamaica and were distinct from the Caribbean islands isolates (Figure 5). Bootstrap support was maintained in this analysis.

All the genotypes clustered in separate branches with high bootstrap support, as previously shown in phylogenetic analysis of other virus sequences [25,17,37]. Our analysis supports the view that the Asian/American strains are phylogenetically different from the main Asian and American genotypes.

### Sequence analysis of Oaxaca isolates

Most of the amino acid (aa) differences were associated with nucleotide changes in the *prM *fragment (13.6%, 6/44 aa). In addition, phylogenetic analysis of *NS3*_484 _showed low variability (1.9%, 2/103 aa). Tables 2 and 3 summarizes the aa changes among the Asian I, Asian II, Cosmopolitan, American and American/Asian Genotype viruses. We compared the aas related to C_91_-prM_1_, NS5_75_, NS5_2206 _and NS3_484 _in all Southeast Asian genotype viruses with American genotypes (Table 2 and 3). The former have greater potential to cause DHF while the latter are associated with DF [18,25]. The Oaxaca isolates in this study and all genotypes with potential to produce DHF contained valine at position 145 in C-prM (prM-31), except for the isolate S707OAX05/MEX, which contained threonine at this position as did in the American genotype. In addition, the Oaxaca isolates in this study and all genotypes with potential to produce DHF contained isoleucine, lysine and glutamine at positions 179, 192 and 818 respectively in NS5. In contrast, the American genotype had valine, arginine and leucine at these positions.

Frequency analysis included in addition to the sequences of Table 2, one sequence with Asian II genotype (M29095) and twelve with Cosmopolitan genotype (AF276619, AF359579, AY858036, AB189122, AB189123, EF440433, DQ448233, DQ448234, DQ448235, DQ448236, DQ448237, AB111454). In addition to the sequences in Table 3, it was included 9 (EU570976, EU570977, EU570978, EU570979, EU570981, EU552540, EU552541, EU552542, EF595827) from Veracruz and 1 (EF595826) from Oaxaca in RdPd domain; and 1(EF591291) from Oaxaca in the Helicase domains.

Interestingly, the Oaxaca isolates in this study and the Caribbean islands strains contained valine at position 106 in C_91_-prM_1 _with a frequency of 0.65 in the Asian/American genotype, while all other genotypes had isoleucine at this position (Table 2). All American/Asian viruses had valine, alanine and glycine at positions 104 (C), 112 (C) and 129 (prM-14), respectively, with a frequency of 1, and isoleucine at position 153 (prM-39) with a frequency of 0.95 because HUAT2/MEX/01 had leucine at this position. The Cosmopolitan genotype had valine and alanine at positions 104 and 112 with a frequency of 0.67 and 0.61, respectively (Table 2). American viruses had lysine and threonine at positions 142 (prM-28) and 145 (prM-31) respectively, with a frequency of 1. Three isolates from Oaxaca 2001 also contained lysine at position 142 (prM-28), resulting in a frequency of 0.075 in the Asian/American genotype. Asian I genotype viruses had serine and isoleucine at positions 101 (C) and 130 (prM-15) with frequencies of 1 and 0.73 respectively. The other genotypes did not contain these amino acids. Cosmopolitan viruses had methionine and asparagine at positions 108 and 143 (prM-29) with a frequency of 0.44 in this genotype. The other genotypes did not contain this aa.

The American/Asian genotype viruses contained isoleucine, lysine, threonine, alanine and valine at positions 155, 748, 762, 810 and 835 in NS5 (Table 3) with a frequency of 1 for positions 155 and 748 and 0.96 for the positions 762, 810 and 835 (Table 3). Asian I contained the same aa at position 762 with a very low frequency of 0.035 in this genotype. Interestingly, our analysis showed that the Asian I genotype, unlike the others, had alanine at position 864 in NS5 rather than threonine with a frequency of 1 (Table 3). Furthermore, the American genotype had valine, arginine, serine and leucine with a frequency of 1 at positions 179, 192, 799 and 818 in NS5; none of the other genotypes has these aas at these positions, resulting in a frequency of 0 (Table 3).

American/Asian and Cosmopolitan genotypes had arginine at position 187 with a frequency of 1 in NS3 (Table 3). Oaxaca isolates had lysine and threonine at positions 249 and 250 in NS3 with frequencies of 0.33 and 0.38. Oaxaca and Veracruz isolates had valine and proline at positions 26 in NS5 and 784 in NS5 with frequencies of 0.33 and 0.56 respectively in the Asian/American genotype. Lysine at position 249 in NS3 may be signature of the Oaxaca strains, and valine and proline at positions 26 and 784 in NS5 may be signatures for the Oaxaca and Veracruz strains.

It is evident that the genotypically different strains contain specific aas that may be used as genotype markers.

## Discussion

The Pan American Health Organization (PAHO) led a campaign to exterminate *Ae. aegypti*, which resulted in the eradication of YF and disappearance of DF in America. *Ae. aegypti *was absent from Mexico in 1947 until 1970. Because the mosquito was not completely exterminated in America and the campaigns were gradually relaxed, *Ae. aegypti *reappeared in most countries. There was a DENV-1 outbreak in Mexico in 1978 and epidemics spread throughout the country. The first reports of outbreaks of DENV-2 were in 1981 and 1984, with 30,000 and 23,000 cases respectively. There were 8 reported DHF cases and 4 deaths associated with DENV-4 in Mexico in 1984. Four DHF cases and one death were reported in 1989 and 2 cases in 1991. DENV-1, -2 and -4 were isolated in 1994, and DENV-3 was reported in 1995. Furthermore, DENV-1, -2, -3 and -4 were isolated in 1997 [34]. This data shows that even when DENV serotypes were co-circulating in Mexico, DHF cases were rare, suggesting that simultaneous circulation of several serotypes (hyperendemicity) is not necessarily sufficient to cause DHF epidemics in the absence of highly virulent strains, as previously reported [18]. Interestingly, the DENV-2 American genotype was replaced by the Asian/American genotype over this period. DF and DHF epidemics increased dramatically in the closing decades of the 20th century, especially in the New World, and Mexico was not excluded [34]. Furthermore, in south Texas (US), all dengue serotypes have circulated periodically, but locally acquired DHF has been recently reported [38]. The causes of this increased incidence of DENV infection, apart from the introduction of the DENV-2 Asian genotype, include demographic, cultural, environmental and political changes. This is clearly shown in Figure 1; DF and DHF cases have increased significantly since 2001 [34]. Furthermore, the proportion of DHF to DF cases has also increased (Figure 1), as in Thailand, where three DENV-2 genotypes have circulated but only viruses assigned to Asian genotype I have been sampled from the population since 1991 [25]. The genotype distribution in Mexico is mostly unknown; consequently, it is very important for epidemiological studies to determine the serotypes and genotypes of the viruses circulating in this country to contribute to the knowledge of geographic distribution, evolution and dispersion of DENV; besides, knowing the virus genotype may allow doctors to judge the risk of DHF to the patient. This may also be useful for laboratory diagnosis, which is an issue of serious concern in all endemic countries; diagnosis may well help in the rigorous follow-up of patients and could save them from the most severe DEN infection (DSS) and death.

Our results showed that DHF was associated with DENV-1 and -2 and one case showed both serotypes (Table 1). Interestingly, we observed no DHF cases associated with DENV-3, as has been shown in epidemiological studies in Mexico [39]. Recently, DENV-3 has been isolated in Brazil but its association with DHF has not been clearly established [40]. In contrast, DENV-3 was associated with DHF in an outbreak in Cuba [13]. Phylogenetic analysis using portions of *C*_91_*-prM*_1_, *NS5*_75_, *NS5*_2206 _and *NS3*_484 _showed no substantial differences (Figures 4, 5).

In this study, we detected the amino acids glutamic acid and valine in place of lysine and threonine at positions 28 and 31 in prM. These aas have been described as specific for the Asian strains correlated with DHF [12]. NS5 also contains lysine 46, arginine 47 and glutamic acid 49 in the MTase domain, essential for the interaction with RdRp required for virus replication and/or infectivity [28]. These three aas are conserved in all sequences of all strains or isolates.

We detected lysine and glutamine at positions 800 and 819 of NS5; these have been reported as markers of the Asian and Asian/American DENV-2 genotypes that have the potential to produce DHF [12]. The interdomain (linker) region 169–179 and the two loops that encircle the entrance of the ATP binding pocket of NS3 [31] were also studied because of the role of this protein in virus replication. This region has also been implicated in the intrinsic flexibility required to couple movements between the NS3 protease and helicase domains [31,32]. We found no change in these amino acids. However, at position 250 in the helicase domain, alanine (apolar) was replaced by threonine (polar) in the isolates in this study and in one strain from China (Table 3). It would seem important to study the structure of this region to determine whether this aa has any role in the enzymatic activity or in maintaining the protein structure.

The NS5 polymerase fragment (737 to 877 residues) contains conserved aas: cysteine at position 847, which participates in the zinc pocket; threonine 790 to aspartic acid 808 and arginine 815 in the priming loop motif; and arginine 737, threonine 794, tryptophan 795 and serine 796, which are involved in GTP binding. However, the aa at position 810 in the priming loop is changed from threonine (polar) to alanine (apolar) in the Asian/American strains including our isolates (Table 3). It would seem important in the future to study the structure around this region to determine the function of this amino acid, particularly whether it has any role in polymerase activity [41].

A dramatic observation was obtained from the analysis of longitudinally sampled data on a temporal scale, where individual lineages or entire clades of viruses frequently arise, persist for a period of time and then disappear [42,43]. All strains analyzed in this work clustered in one branch with the Asian/American strains. Previously, in a survey during 2001 by the Department of Health [17,33], we reported this genotype in isolates from Oaxaca on the basis of the protein C sequence and a fragment of the prM gene (438–572). The finding of the same Asian/American genotype in the isolates from the 2005 and 2006 outbreaks indicates that they circulated and became prevalent in Oaxaca and Veracruz States; however, the isolates in this study were grouped with the strains from the Caribbean islands, and the 2001 isolates with the Jamaica strain, with a high consistency of 94%. Also, these 2005–2006 isolates from Oaxaca and Veracruz showed valine in position 106 in prM, which was also shown by the Caribbean islands strains. These isolates also clustered with the Caribbean islands strains Cuba 97, Dominican Republic 2001 and Martinique 98 in the phylogenetic analysis of NS5_75 _and NS3_484 _(results not shown), supporting this view of their geographic origin (Figure 2). All trees clearly distinguish among the five genotypes of DENV-2, as reported previously [24,25], with strong bootstrap support (Figures 4, 5). Little is known of the history of this American/Asian signature circulating in the Caribbean islands. However, Rodriguez-Roche et al. [44] found that clinical severity increased over time during the epidemics expressed by a higher ratio DHF to DF for the Cuban strains.

Apparently, there has been a clade replacement of the American genotype for the Asian/American genotype originated in Southeast Asia in Oaxaca (2001 outbreak) and lately that strains were substituted for the Oaxaca strains (2005–2006 outbreaks) with the probable origin in the Caribbean islands, possibly Cuba (1997 outbreak). Furthermore, the geographic origin of the Veracruz strains may be also the Caribbean islands.

Since analysis of the 98 C-prM sequences showed that all Asian/American viruses had valine, alanine, glycine and isoleucine at positions 129 (prM-14) and 153 (prM-39) with a frequency of 1, while none of the other genotypes had these aas, they may be used as signatures of this genotype. The signature for American genotype viruses may be threonine at position 145 (prM-31) since none of the other genotypes had this amino acid. The signature for the Asian I genotype viruses may be serine at position 101 (C) with a frequency of 1.

From the 65 (MTase) and 76 (RdRp) sequences analyzed, all Asian/American genotype viruses contained isoleucine, lysine, threonine, alanine, lysine and valine at positions 155, 748, 762, 810 and 835 (Table 3) and none of the other genotypes had these aas. Our analysis also showed that the Asian I genotype, unlike the others, had alanine at position 864 (RdRp). The American genotype had valine, arginine, serine and leucine with frequency of 1 at positions179, 192, 799 and 818, but none of the DENV-2 genotypes had it. All aas specific for a genotype may be used as signatures for that genotype. Although fewer sequences were analyzed than for C-prM, because these genes have been less studied and consequently fewer sequences have been reported. The Asian 2 and Cosmopolitan genotypes showed no specific signature in the present study. This may be attributable to the broad spectrum of the Cosmopolitan genotype strains and the low number of Asian 2 sequences reported.

Lysine at position 249 in NS3 may be signature of the Oaxaca strains, and valine and proline at positions 26 and 784 in NS5 may be signatures for the Oaxaca and Veracruz strains, since none of the other genotypes or strains from the Asian/American genotype had these aas.

Some of the observed changes in the viruses could be due to mosquito adaptation as well as to the vertebrate hosts, since there is evidence that strains of DENV-2 may differ in their ability to infect *Ae. aegypti *[45], perhaps through specific virus-cell receptor interactions [46,47] by strong selection, or competition between DENV serotypes [48]. It has been suggested that the Asian genotype may spread more readily because it infects *Ae. aegypti *mosquitoes more efficiently than the American genotype [45].

The amino acid changes between the isolates of this study and those from 2001 may be due to a high mutation rate [49] or recombination [50] contributing to the generation of new biologically successful strains.

Phylogenetic analysis of the C_91_-prM_1_, NS5_75_, NS5_2206 _and NS3_484 _sequences in the DENV-2 isolates demonstrated that the Asian/American genotype is circulating in Oaxaca and Veracruz, showing specific amino acid changes that may be used successfully as genotype markers. Differences in the NS5_2206 _tree, which showed that the isolates in this study clustered with the strains from the contained American genotype, may be due to the low variability of this domain. In addition, this analysis shows a clear correlation between genetic diversity and geographical location. As has been suggested previously [43,51], viral dispersion may play a significant role in generating new varieties and effects on people who contract the illness.

## Conclusion

We have demonstrated that DENV-1, -2 and -3 are circulating in Oaxaca and DENV-1, -2, -3 and -4 in Veracruz (2006), resulting in many DHF cases (Figure 1, 3). Eight DHF cases were associated with DENV-1, 27 with DENV-2 and one case with both DENV-1 and -2. The origin of the isolates in this study appears to be linked to the signature from the Caribbean islands genotypes. Our results confirm that DHF is endemic in Oaxaca and Veracruz States and that the presence of the DENV-2 Asian/American genotype and DENV-1 contributes to the increased number of DHF cases, a finding linked to the epidemiological data showing an increase in the ratio of DHF to DF cases in 2005–2006. We also identified amino acids encoded in C_91_-prM_1_, NS5_75_, NS5_2206 _and NS3_484 _that are specific to the Asian/American strains; and one amino acid specific to the isolates of this study and the Caribbean islands strains. These aas may be used as markers of genotypes and geographic origins.

## Methods

### Viruses

The strains of DENV used in this study were: DENV-1 Hawaii; DENV-2 New Guinea C (NGC); DENV-3 H-87; and DENV-4 H-341. These strains were provided by Dr. D. J. Gubler (Division of Vector-borne Infectious Diseases, Center for Disease Control, Fort Collins, CO, USA) and kindly provided by Dr. Blanca Ruiz (Biomedicas, UNAM). Isolates of DENV-2 viruses were obtained from acute-phase plasma collected from patients with DF or DHF by the Secretaria de Salud in Oaxaca, Mexico during 2005–2006, and by the Instituto Mexicano del Seguro Social (IMSS) during 2006. Twenty-nine isolates were kindly provided by the State of Oaxaca Public Health Laboratory and 65 by the IMSS in Veracruz. These were anonymous samples; only information about the clinical disease associated with the respective infection was provided (Tables 1).

### DENV infected cells and virus isolation

*Ae. albopictus *clone C6/36 cells were grown at 28°C. After 18 h of culture, cells (2 × 10^6^/100 mm plate) were infected with 0.2 ml DENV-2 inoculum with an input MOI of 600 PFU/cell and were incubated at 28°C for 10 days.

Viruses were isolated as previously described [17] with a few modifications. After 18 h of culture, C6/36 cells (10^6^/15 ml tube) were infected with 0.01 to 0.1 ml of serum specimen per tube, diluted to 1 ml with medium, and incubated for 2 h at 28°C. After one wash, 3 ml MEM was added and the cells were cultivated for approximately 15 days at 28°C (passage number 1). Cells were observed every day and when a cytophatic effect was apparent from syncytium formation and cellular lysis, the cells were harvested and centrifuged at 3000 rpm for 5 min. The pellet was suspended in 0.6 ml of MEM and stored in aliquots of 0.15 ml at -70°C. The supernatant (approximately 2.5 ml) was stored in 2 aliquots of 1 ml and one of 0.5 ml at -70°C. To obtain passage numbers two and three, C6/36 cells were incubated with 1 ml of the supernatant obtained from the first or second passage for 2 h at 28°C and the same procedure described above was followed. Serotypes in all samples were determined in the isolates obtained from the first, second or third culture passages.

### RNA extraction

Total RNA was extracted from cell culture supernatant using Trisol^® ^LS (Gibco BRL., Gaithersburg, Md.) according to the manufacturer's recommendations. Ethanol-precipitated RNA was recovered by centrifugation and air-dried. The RNA pellet was suspended in 50 μl water treated with diethylpyrocarbonate (DEPC, Sigma-Aldrich) and used as template for Reverse Transcription with the Polymerase Chain Reaction (RT-PCR).

### Reverse transcription-polymerase chain reaction (RT-PCR)

The RT-PCR protocol described by Seah et al. [52] was followed to determine the DENV serotype. Reaction mixtures were stored at -20°C pending further processing.

Synthetic oligonucleotide primer pairs were designed on the basis of published sequence data for DENV strains 16681, New Guinea C and Jamaica 1409 to amplify and partially sequence the following genes: *protein C *from nucleotides 91 to 351(C_91_); *prM *from nucleotides 1 to 123 (prM_1_) [17]; *NS5 *from nucleotides 73 to 588 (NS5_73_) and 2206 to 2613 (NS5_2206_); and *NS3 *from nucleotides 484 to 786 (NS3_484_). All assays were performed using the ThermoScript™ RT-PCR System containing PlatinumR Taq (Invitrogen, Life Technologies). A mixture of 5 μl total RNA (0.1–0.5 μg), 50 ng of hexamers/reaction and DEPC-treated water (total volume 50 μl) was incubated at 85°C for 5 min and chilled on ice. The first extension was carried out at 25°C for 10 min and then at 50°C for 70 min. The PCR reaction was carried out by incubation of 50 pmol of the corresponding sense and antisense PCR primers, 2 μl of the cDNA synthesis reaction mixture and 2.4 mM magnesium sulfate as per the manufacturer's recommendations.

To amplify the NS5 gene we used the sense primer from nucleotides 7398–7413 (CAT GGG CNY TNT GYGA) and the antisense primer from nucleotides 10551–10570 (GGA GGG GTC TCC TCT AACC). The PCR conditions for amplifying the 2700 bp fragment for NS5 were: 95°C for 5 min, 30 cycles of 94°C for 1 min, 49°C for 30 s, 72°C for 2 min 30 s, and final extension of 72°C for 10 min followed by storage at 4°C.

### Sequencing of PCR fragments

For automated sequencing, spin column-purified (Quiagen, Chatsworth, CA.) DNA fragments were analyzed by the cycle-sequencing dye terminator method. A Big Dye Terminator Cycle Sequencing Ready Reaction Kit (ABIPRISM 100, Applied Biosystems, Foster City, CA) was chosen and sequencing was conducted using an Applied Biosystems Prism 310 in a short capillary (47 cm by 50 μm inside diameter), and Performance Optimized Polymer 6 (Perkin-Elmer, Applied Biosystems).

### Phylogenetic analyses

Neighbor-joining (NJ), maximum parsimony and minimal evolutionary [53] analysis methods were used to reconstruct phylogeny. Distance with gamma correction was generated by Neighbor-joining, maximum likelihood and Tamura and Nei [54] evolutionary models. Support for the derived phylogenies was examined with bootstrapping over 1000 replications. For all DENV-2 analyses, DENV-1, -3 and -4 were used as outgroups. All these analyses were performed using MEGA V4 [55].

## Authors' contributions

CEGG carried out the RT-PCR assays using RNA isolates from passages 1, 2 and 3 to determine serotype and to amplify the NS5 and NS3 genes for genotyping, and co-wrote the manuscript. GPR obtained the isolates and carried out the RT-PCR assays using RNA from passages 1, 2 or 3 to sequence the C-prM gene for genotyping by phylogenetic analysis. CEGG, GPR and MLM participated in the discussion of results, helped to review the manuscript and assisted with the literature validation. JNE and RRL provided the serum samples from Veracruz and the clinical data corresponding to each patient, and helped in obtaining the isolates. FJR, LRRP and AC collected the patient serum samples from Oaxaca, Mexico, and helped to obtain the isolates and clinical data from Oaxaca. MCN participated in the discussion of results and reviewed the manuscript. MLM proof-read and assembled the manuscript. All authors participated in the discussion of results and read and approved the final manuscript.
